# MeCP2 Dependent Heterochromatin Reorganization during Neural Differentiation of a Novel *Mecp2*-Deficient Embryonic Stem Cell Reporter Line

**DOI:** 10.1371/journal.pone.0047848

**Published:** 2012-10-24

**Authors:** Bianca Bertulat, Maria Luigia De Bonis, Floriana Della Ragione, Anne Lehmkuhl, Manuela Milden, Christian Storm, K. Laurence Jost, Simona Scala, Brian Hendrich, Maurizio D’Esposito, M. Cristina Cardoso

**Affiliations:** 1 Department of Biology, Technische Universität Darmstadt, Darmstadt, Germany; 2 Institute of Genetics and Biophysics “A Buzzati Traverso”, Naples, Italy; 3 Istituto di Ricovero e Cura a Carattere Scientifico Neuromed, Pozzilli, Italy; 4 Wellcome Trust - Medical Research Council Stem Cell Institute and Department of Biochemistry, University of Cambridge, Cambridge, United Kingdom; Université Paris-Diderot, France

## Abstract

The X-linked *Mecp2* is a known interpreter of epigenetic information and mutated in Rett syndrome, a complex neurological disease. MeCP2 recruits HDAC complexes to chromatin thereby modulating gene expression and, importantly regulates higher order heterochromatin structure. To address the effects of MeCP2 deficiency on heterochromatin organization during neural differentiation, we developed a versatile model for stem cell *in vitro* differentiation. Therefore, we modified murine *Mecp2* deficient (*Mecp2* ^−/y^) embryonic stem cells to generate cells exhibiting green fluorescent protein expression upon neural differentiation. Subsequently, we quantitatively analyzed heterochromatin organization during neural differentiation in wild type and in *Mecp2* deficient cells. We found that MeCP2 protein levels increase significantly during neural differentiation and accumulate at constitutive heterochromatin. Statistical analysis of *Mecp2* wild type neurons revealed a significant clustering of heterochromatin per nuclei with progressing differentiation. In contrast we found *Mecp2* deficient neurons and astroglia cells to be significantly impaired in heterochromatin reorganization. Our results (i) introduce a new and manageable cellular model to study the molecular effects of *Mecp2* deficiency, and (ii) support the view of MeCP2 as a central protein in heterochromatin architecture in maturating cells, possibly involved in stabilizing their differentiated state.

## Introduction

Heterochromatin is defined as chromatin that stays densely packed during interphase. Cytologically, heterochromatin can be further subdivided into constitutive and facultative heterochromatin [1], [2], with facultative heterochromatin usually differing between cell types [3]–[6]. Hence, heterochromatin is amongst the key features of cellular differentiation and transdifferentiation [7], [8].

Heterochromatin is commonly associated with transcriptional silencing and characteristic epigenetic marks on the level of histone [9] and nucleotide modifications [10]. Together both kinds of marks act on the degree of compaction and accessibility of DNA, thereby creating sub-nuclear compartments of less dense euchromatin and more compacted heterochromatin. In mouse cells the majority of constitutive heterochromatin is constituted by AT rich tandem repeats (major satellites) adjacent to the centric region of the chromosomes [11]. It is well known that pericentric heterochromatin domains of different chromosomes are organized in so called chromocenters [12], [13] during interphase which reorganize during cellular differentiation [5], [6], [14].

One characteristic epigenetic mark of chromocenters is the covalent methylation at the carbon 5 position of cytosine (5 mC) in CpG dinucleotides. This epigenetic mark is read and interpreted by the methyl cytosine binding protein 2 (MeCP2) [15]–[18]. The *Mecp2* gene is encoded on the X chromosome [19] and its product was initially identified as a selective 5-methyl cytosine binding protein [20]–[22]. Meanwhile it is one of the best-studied members of the methyl cytosine binding protein (MDB) family [23]–[27] and was found to be mutated in the neurological disorder Rett syndrome (RTT, OMIM #321750) occurring with a frequency of 1 in 10,000 female birth [28]–[31]. Children affected by RTT show an apparently normal development up to 6–18 months. Thereafter they start to lose acquired hand skills and spoken language and instead of further progress most RTT patients develop repetitive hand movements, autistic features, seizures and abnormalities in growth, breathing and sleep [29].

The devastating effects of RTT were originally considered to be the consequence of *Mecp2* deficiency in adult neurons resulting in gene deregulation [32]. This hypothesis was supported by deleting *Mecp2* only in neurons, which resulted in RTT-like symptoms in mice [33], [34]. A variety of studies suggested that MeCP2 acts as a transcriptional repressor [22], [35]–[39] exhibiting increasing protein levels with progressive *in vivo* and *in vitro* differentiation [5], [40], [41]. Surprisingly, analysis of gene expression profiles in the hypothalamic and cerebellar regions of *Mecp2*-null and overexpressing mice [42], [43] suggested that MeCP2 acts as a transcriptional activator for several thousand of genes. Although the differences in gene expression were subtle, the notion of MeCP2 as an activator of transcription was supported by its biochemical interaction with the transcriptional activator CREB [42]. As MeCP2 was recently reported to be expressed in neurons at near nucleosome levels tracking methylated CpG dinucleotides and replacing histone H1 [41], it might function as a global chromatin architect. *Mecp2* deficiency seems also to affect glial cells with not yet fully understood consequences for neural cell survival [44]–[47]. Several lines of evidence show that not only the lack of functional MeCP2 but also MeCP2 protein oversupply results in severe symptoms [48]–[50]. Overall, the accumulating evidence argues for a multifunctional role of MeCP2 acting as a modulator of gene expression levels [35], [37], [42], [43], [51], [52] and, together with several other proteins [14], [37], [53], as a global heterochromatin organizer [5], [41], [54], [55] thereby stabilizing a cell’s differentiated state.

Here we focus on heterochromatin reorganization, which is a common feature of cellular differentiation in a variety of eukaryotic cells [3]–[5], [55]–[58]. Previously it has been shown that MeCP2 protein is necessary and sufficient for chromatin clustering and that ectopic MeCP2 is able to mimic heterochromatin reorganization [5], [54]. Given that lack or malfunction of MeCP2 severely affects brain function we were interested in MeCP2 dependent heterochromatin reorganization during neural differentiation. Hence, we established a new murine *Mecp2* deficient (*Mecp2* ^−/y^) embryonic stem (ES) cell line as a part of a versatile and easy to handle *in vitro* cellular differentiation system. A feeder free, one-step differentiation protocol [59] allowed us to follow specifically neural differentiation of *Mecp2* wild type (*Mecp2* ^wt^) and deficient (*Mecp2* ^−/y^) cell cultures. We analyzed and quantified heterochromatin organization in *Mecp2* ^wt^ cells during neural differentiation and compared the results to *Mecp2* deficient cells. Although the latter were able to differentiate, the absence of MeCP2 led to significant differences in their chromatin higher order organization.

## Materials and Methods

### Generation and Characterization of *Mecp2* ^−/y^ *tau::EGFP (Mecp2* ^−/y^ tEG) Stem Cells

*Mecp2* deficient (*Mecp2* ^−/y^) ES cells were made by Cre-mediated deletion of a conditional *MeCP2* allele in the ES cell line described in Guy *et. al.* [34]. An *EGFP* knock-in into the *Mapt/tau* locus was created using a vector kindly provided by Yves-Alain Barde (Basel, Switzerland) as described [60] (Figure S1A). Properly targeted clones were identified by PCR with primers surrounding *Mapt/tau* exon 1 (forward: 5′ *AGGACCTAGCCAGCTGTGAA*; reverse: 5′ *GAACTTCAGGGTCAGCTTGC*). To verify properly targeted clones an inverse PCR approach was performed [61]. Specifically, 1 µg genomic DNA was treated with 10 U *BamH* I (New England Biolabs, USA), purified, and subjected to a DNA ligation reaction. Inverse PCR was subsequently performed with purified ligated products using the following primers: forward 5′ *CTCAGGCAACACTTAAACTC*; reverse 5′ *TCAGATCACTAGACTCAGCA* (Figure S1B). For the following experiments one verified clone has been used and checked for genomic stability by karyotyping (Figure S1C).

### Stem Cell Culture and Differentiation

Murine wild type *Mecp2* TK 23 *tau::EGFP* embryonic stem cells were kindly provided by the Austin Smith lab (Cambridge, UK) [60], [62], [63]. Both, *Mecp2* TK23 *tau::EGFP* wild type and *Mecp2* deficient cells, hereafter referred to as MeCP2^wt^ tEG and *Mecp2* ^−/y^ tEG, were originally derived from the murine strain E14 [34] and cultured feeder-free at 37°C and 5% CO_2_ either on gelatin coated culture vessels or poly-D-lysine/laminin coated glass slides. Standard gelatin coating was performed using a filter sterilized 0.1% (v/v) gelatin solution (Sigma-Aldrich, Germany) in sterile PBS for at least 30 min at room temperature. Glass slides were coated by incubation in 0.01 mg/ml poly-D-lysine (Sigma Aldrich, USA) for 30 min at 37°C, followed by two brief washing steps in 1× PBS. Subsequently, slides were incubated over night in 2 µg/ml laminin (Sigma-Aldrich, Germany) in PBS at room temperature.

Undifferentiated stem cells were maintained in expansion medium consisting of: Glasgow minimal essential medium (GMEM, Sigma-Aldrich, Germany) substituted with 2 mM glutamine (Life technologies, Germany), 1 mM sodium pyruvate (Sigma-Aldrich or Life Technology, both Germany), 100 µM non essential amino acids (Sigma-Aldrich, Germany), 10% (v/v) FCS (PAA, Germany), 0.05 mM 2-mercaptoethanol (Carl Roth or Sigma-Aldrich, both Germany), 100 U/ml penicillin/streptomycin (Life Technology, Germany), and 1,000 U/ml leukemia inhibitory factor (LIF, Millipore, USA) or Esgro LIF (Sigma-Aldrich, Germany) respectively. Expansion medium was exchanged in 24 hour intervals and cultures were passaged every second day.

For differentiation according to Fico *et al.* [59] 10^3^ to 2.9×10^5^ cells/cm^2^ were seeded on either gelatin or poly-D-lysine/laminin coated glass slides (see above) and maintained 24 hours in expansion medium. To compensate for the slower growth of *Mecp2* ^−/y^ tEG, the cell number seeded was doubled compared to *Mecp2* ^wt^ tEG cultures. Differentiation was induced by LIF deprivation in LIF-free Knockout Dulbecco’s minimal essential medium (Life technology, Germany) supplemented with 15% knockout serum replacement (Invitrogen, Germany), 2 mM glutamine (Life technologies, Germany), 100 U/ml penicillin/streptomycin or 50 µg/ml gentamicin (Sigma-Aldrich, Germany), and 0.1 mM 2-mercaptoethanol (Carl Roth or Sigma-Aldrich, both Germany). During differentiation, medium was changed every day and cells were fixed for immunostaining at day 0, 7, 13, 21, and 23 (whereas day 0 refers to undifferentiated cells before LIF deprivation).

### Immunoblotting

Proteins were detected using the following primary antibodies: anti-beta actin (as loading control) 1∶2,500 (A2066, Sigma Aldrich, Germany) and anti-MeCP2 1:2,000 (M9317, Sigma Aldrich, Germany). Signals were visualized using a goat anti-rabbit IgG-HRP secondary antibody (sc-2004, Santa Cruz, USA) in a 1∶10,000 dilution.

### Immunofluorescence

For immunofluorescence cells were cultured on gelatin or poly-D-lysine/laminin coated glass slides. If not stated otherwise all incubation and washing steps were performed at room temperature for 5 min. Neuronal cells were identified by *tau* promoter driven EGFP reporter signals and further characterized with specific marker antibodies for neurons and astroglia. DNA and chromocenters were visualized with 4′,6 diamidino-2-phenylindole (DAPI, 1 µg/ml, Sigma-Aldrich, Germany) and the presence or absence of MeCP2 was shown with the monoclonal rat anti-MeCP2 clone 4H7 [64]. For all immunofluorescence experiments in combination with the anti-MeCP2, cells were fixed in 4% PFA in PBS (EM grade, Electron Microscopy Science, USA) for 10 min at 4°C or 30 min at room temperature. After fixation, samples were washed once in PBS and permeabilized for 10 min in PBS/0.25% TritonX-100 (Sigma-Aldrich, Germany). Following permeabilization and two to three washing steps in PBS/0.02% Triton X-100/0.02% Tween 20 (Sigma-Aldrich, Germany) samples were incubated for 15–30 min in blocking solution (4% BSA in PBS/0.02% Tween 20 or PBS/0.1% Triton X-100/10% normal goat serum). Primary antibody incubation was done over night at 4°C or one hour at room temperature. Antibodies used are listed in Table 1.

**Table 1:** List of antibodies used.

Antibody	Host	Dilution	(Catalog no.) Company/Reference
anti-Tyrosine Hydroxylase (TH)	mouse	1∶100	MAB 1637, Merck Millipore, Germany
	rabbit	1∶200	AB152, Merck Millipore, Germany
anti-Serotonin (Sero)	rabbit	1∶200	S-5545, Sigma Aldrich, Germany
anti-Glial Fibrillary Acidic Protein(GFAP)	rabbit	1∶500	AB 5804 Merck Millipore, Germany
	rabbit	1∶300	Z0334Dako Cytomation Denmark
anti-beta Tubulin III (ßTubIII)	mouse	1∶100	MAB 1637, Merck Millipore, Germany
anti-MeCP2	rat	undiluted	Jost et al. [64]
anti-mouse IgG-Cy3	donkey	1∶100	715-165-151, Jackson Immuno Research, USA
anti-mouse IgG-Cy5	donkey	1∶100	715-175-450, Jackson Immuno Research, USA
anti-rat IgG-Cy5	donkey	1∶100	712-175-153, Jackson Immuno Research, USA
anti-rat IgG-Cy3	donkey	1∶100	712-165-153, Jackson Immuno Research, USA
anti-rabbit IgG-Cy3	donkey	1∶100	711-165-152, Jackson Immuno Research, USA

After three washing steps in PBS, samples were DAPI stained (333 ng/ml, Sigma-Aldrich, Germany) for 10 min. Prior to mounting in 90% glycerol in 20 mM Tris-HCl pH 7.4 supplemented with 1,4-diazobicyclo [2,2,2]-octane (DABCO, Sigma-Aldrich, Germany), slides were washed in PBS and briefly dipped into distilled water to remove excess salts.

**Figure 1:**
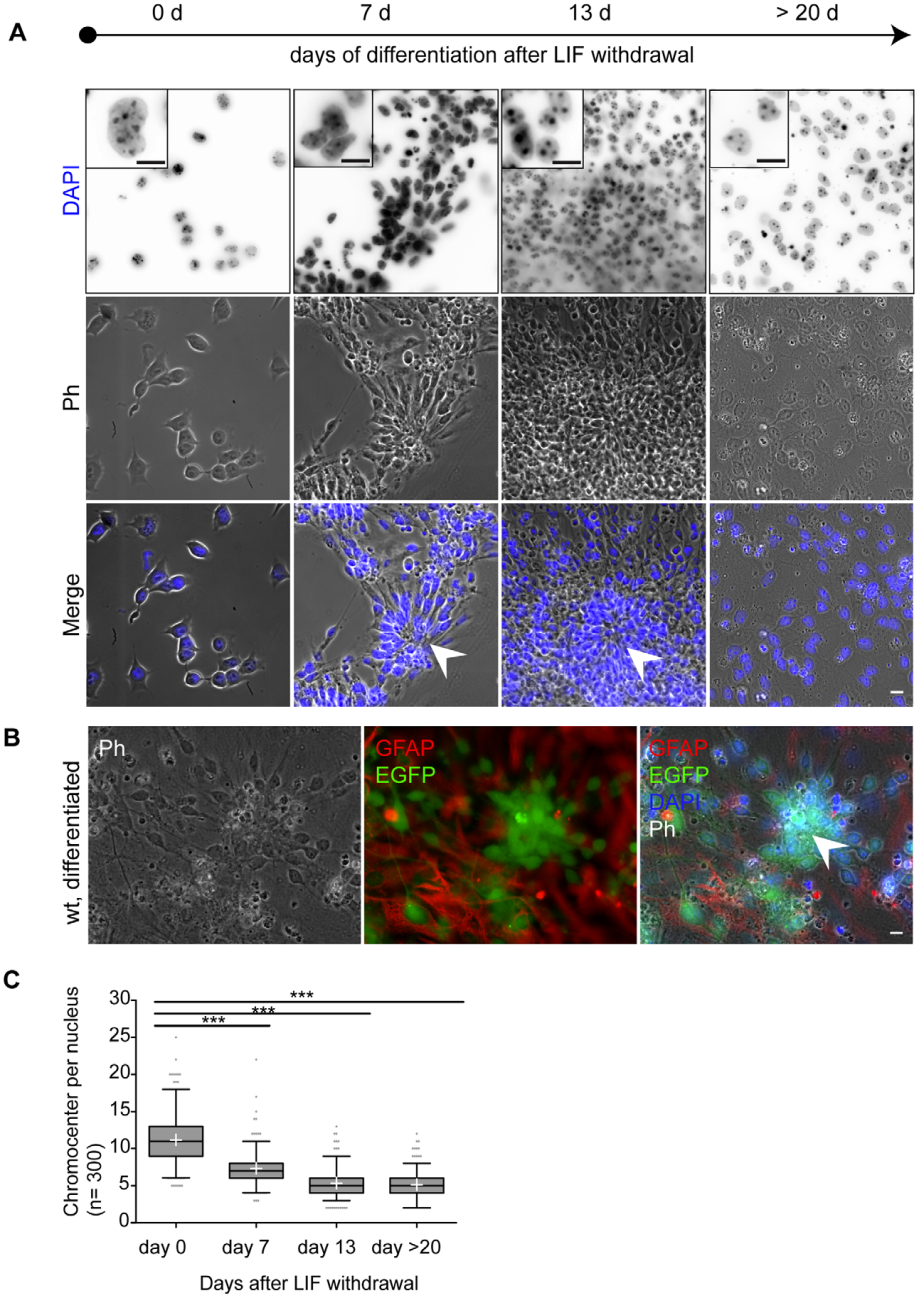
*In vitro* differentiation of *Mecp2* wild type ES cells is accompanied by large scale heterochromatin reorganization. A. Shown are representative low and high (insets) magnification images of phase contrast (Ph) and DAPI DNA staining. Cells were kept in an undifferentiated state (day 0) in LIF containing medium. Differentiation of ES cells was induced by LIF withdrawal and plating into differentiation medium. Rosettes (arrow heads) indicated regions of neural differentiation, clearly visible as of day 7. Shortly thereafter EGFP positive neurons could be detected around and within rosettes and mark differentiating neural cells. At day 13 cells exhibit a tissue like growth. Bar: 10 µm. B. The neural identity of rosettes could be demonstrated by *EGFP* reporter expression driven by the neural *tau* promoter as of day 5 to 7 and most prominent after 13 days. Most EGFP positive cells are located within rosette structures, often surrounded by GFAP positive astroglia. Bar: 10 µm. C. Replicas of 100 DAPI stained nuclei were statistically analyzed and shown combined in a whisker box plot. Whiskers depict the 5–95 percentile of the confidence interval; the median is shown as a horizontal line within the box, and mean values are highlighted as white crosses. We noticed a highly significant (p<0.0001) decrease of mean chromocenter number per nucleus within the first two weeks of differentiation. Thereafter, the chromocenter number did not change significantly (p = 0.177). Statistical significance was tested with individual unpaired t-tests following Welch’s correction.

### Microscopy

Epifluorescence and phase contrast images were obtained on a Zeiss Axiovert 200 microscope equipped with Plan-Apochromat 63×/NA 1.4 (pixel size XY = 104 nm) and Plan-Neofluar 40×/NA 1.3 (pixel size XY = 168 nm) oil immersion objectives and a Zeiss AxioCam mRM camera.

For acquisition of 3 D and multicolor confocal images we used a Perkin Elmer UltraVIEW VoX spinning disk confocal system mounted on an inverted Nikon Ti-E microscope. Images were taken with a Hamamatsu C9100-50 EMCCD camera and Nikon CFI Apochromat TIRF 60 x/NA 1.49 (pixel size XY = 120.478 nm) or CFI Plan Fluor 40×/NA 1.3 (pixel size XY = 163.2 nm) oil immersion objectives.

**Figure 2:**
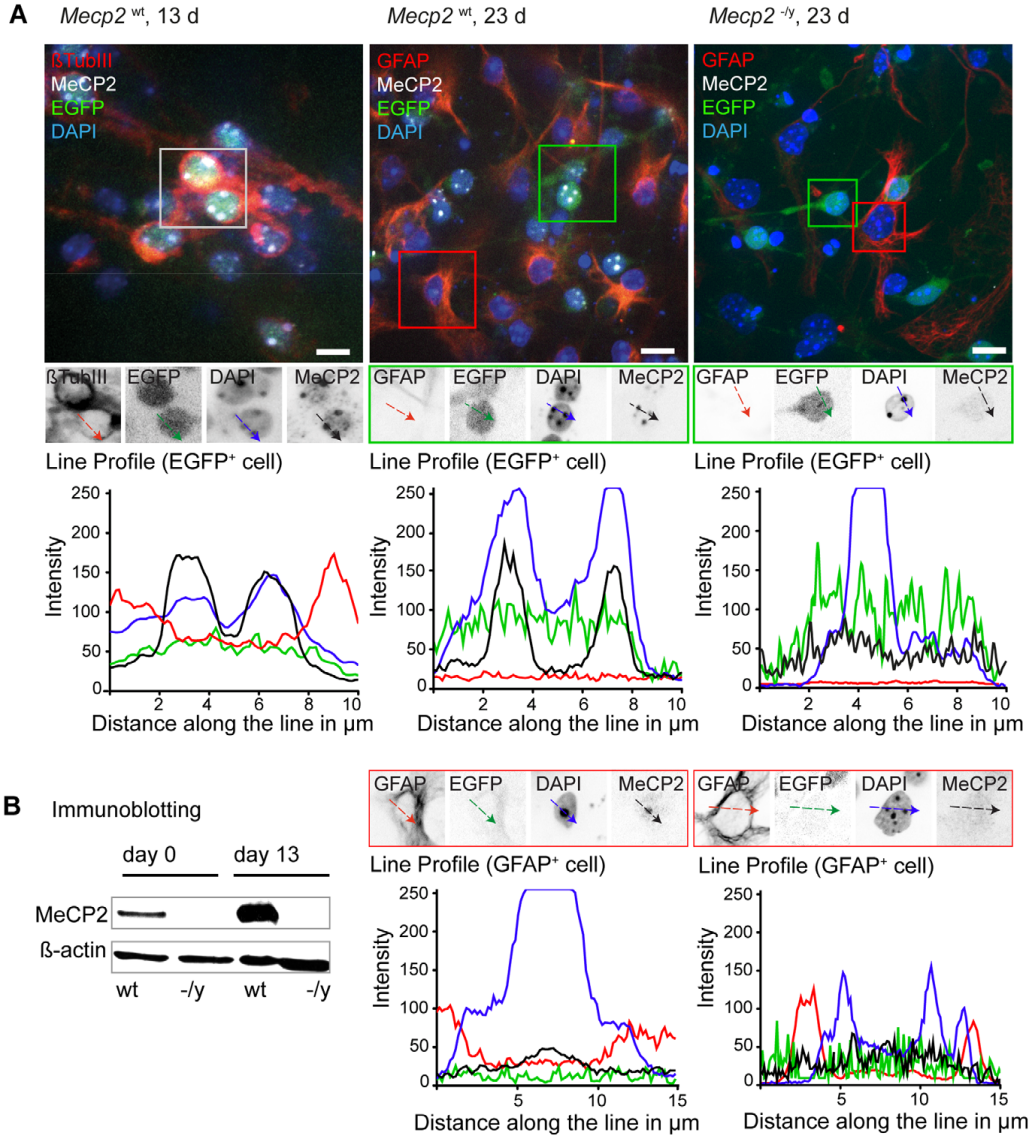
MeCP2 becomes detectable in heterochromatic chromocenters at late differentiation stages. A. *Mecp2* ^wt^ and *Mecp2* ^−/y^ tEG cells were differentiated, fixed, and immunostained with antibodies to MeCP2. *Tau* promoter driven *EGFP* expression highlights neuronal cells. Additional markers as beta tubulin III (neuronal cell) and GFAP (astroglia) allow discrimination of different cell populations. In immunofluorescence experiments MeCP2 protein is detected as of day 13 (left) and remains constant thereafter (middle) in EGFP^+^ cells. Line profiles across chromocenters highlight MeCP2 protein accumulation at these structures (black line) as well as their intense DAPI signal (blue line). Some GFAP^+^ astroglia revealed a relative weaker MeCP2 signal (black line) compared to neurons. *Mecp2* deficient cells (right) were used as control. Bar: 10 µm. B. Immunoblot experiments demonstrate the increase of MeCP2 protein over time in wild type cells (wt) while *Mecp2* deficient cells (−/y) show no signal. Beta actin was used as a control for equal loading.

### Image Analysis

For chromocenter evaluation multichannel Z–stacks were acquired with a step size of 0.5 µm (channels first than Z). We compared *Mecp2* ^wt^ tEG to *Mecp2* ^−/y^ tEG stem cells and evaluated chromocenter numbers at selected time points of differentiation (undifferentiated cells, days 7, 13, and 21 or 23 after LIF withdrawal and plating in differentiation medium). Z-stacks were open in Image J (http://rsbweb.nih.gov/ij/), and in case of multiple channel images, one window per channel was used and windows were synchronized. The number of chromocenters per nucleus was then counted manually. Whenever possible, chromocenters were visualized by anti-MeCP2 and DAPI signals and counted in all cells of interest. In case of undifferentiated cells, early time points, and *Mecp2* deficient cells chromocenters were evaluated by DAPI signals alone, if possible in combination with additional markers.

**Figure 3:**
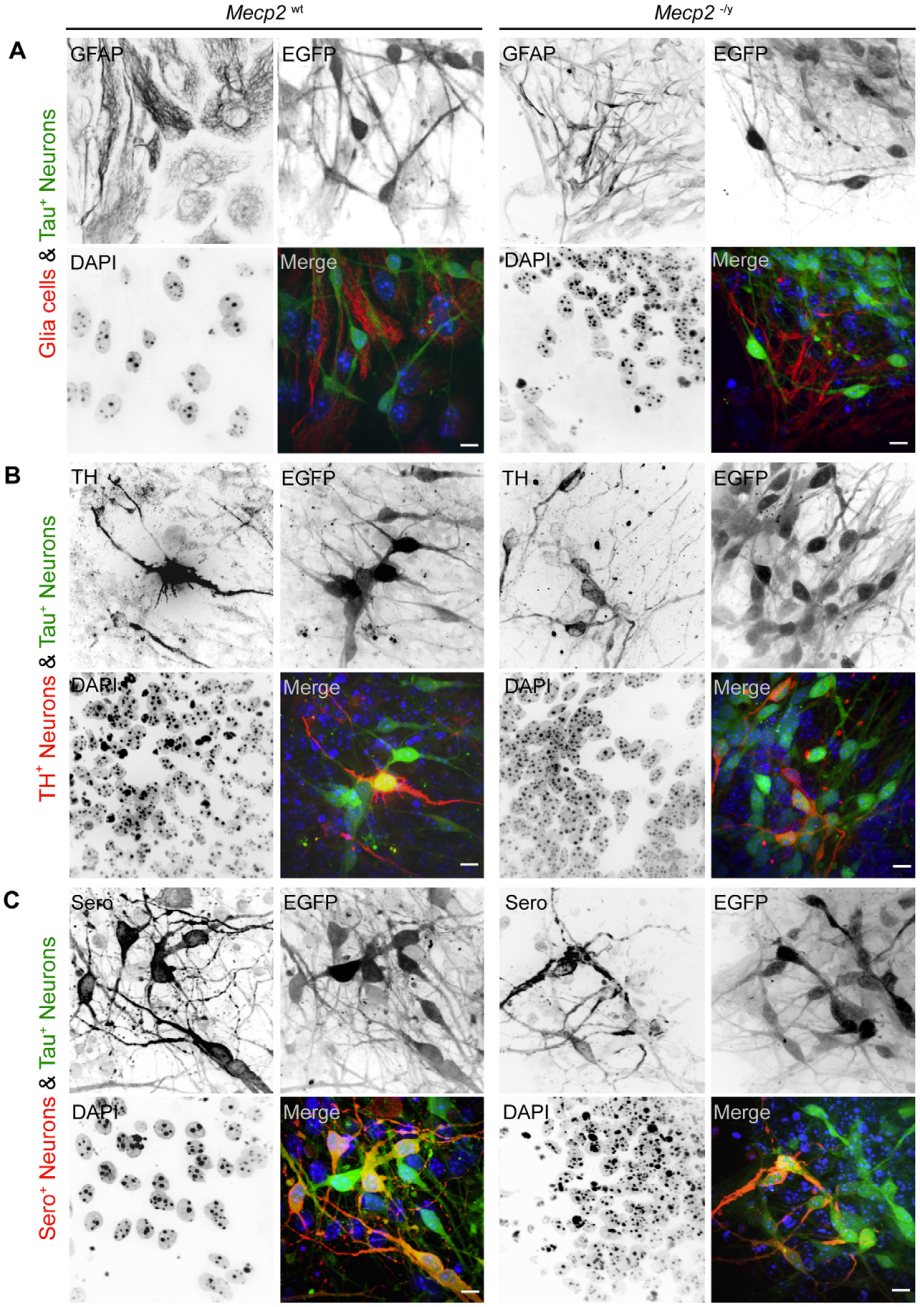
MeCP2 absence does not prevent neuronal and astroglia differentiation. Confocal microscopy optical sections of *Mecp2* wild type (left) and *Mecp2* deficient cells (right) at differentiation day 13 revealed astroglia (GFAP^+^) and different neuronal subtypes (EGFP^+^, TH^+^, Sero^+^). Bar: 10 µm. A. Differentiated *Mecp2* wild type and *Mecp2* deficient cells exhibit GFAP positive astroglia (red) and EGFP positive neurons (green). Both labels marked mutually exclusive cell populations. B. The population of EGFP positive cells could be further subdivided into tyrosine hydroxylase (TH^+^) positive neurons (red). Those cells were found to be present in both *Mecp2* wild type and *Mecp2* deficient cells. While not all EGFP^+^ neurons (green) are TH^+^, all TH^+^ are positive for EGFP (yellow color in overlay). C. In addition to a TH^+^/EGFP^+^ subpopulation neurons, another serotonin positive (Sero^+^) subpopulation was detected in both cell lines. Similar to the situation described above, Sero^+^ cells (red) were always EGFP^+^ (green) while a considerable amount of EGFP^+^ cells were Sero^−^.

For what we refer to as “unbiased” counting approach, three biological replicates of 100 nuclei each were evaluated per time point in *Mecp2* ^wt^ tEG *Mecp2* ^−/y^ tEG cells. At day 0 (undifferentiated cells) nuclei were selected at random and at later time points (day >20, day 13 and 7) evaluation was focused on cells with neural morphology or within neural rosettes.

For population specific chromocenter evaluation, anti-GFAP positive cells or neurons positive for GFP and a population specific marker (either anti-serotonin or anti-tyrosine hydroxylase positive cells) were selected at day 13 and evaluated as described.

Co-localization of MeCP2 and DAPI signals was analyzed on individual optical sections within a Z-stack by line profiling in ImageJ.

**Figure 4:**
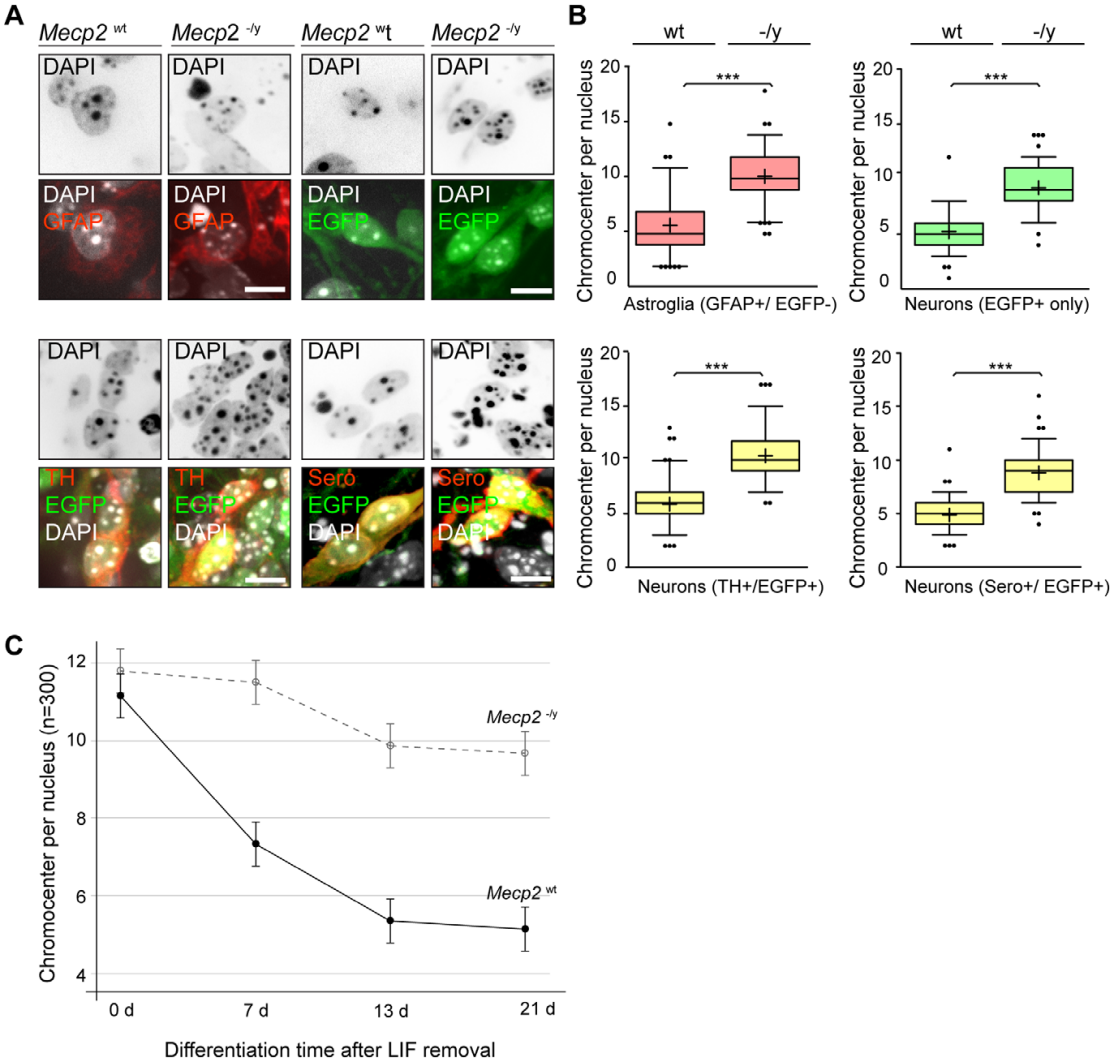
*Mecp2* deficient neurons and astroglia cells have significantly more chromocenters than wild type cells. A. Representative confocal microscopy optical sections of differentiated *Mecp2* wild type and *Mecp2* deficient nuclei revealed characteristic differences in heterochromatin organization. Positive immunostaining for the astroglial marker GFAP (upper left) or for neuronal markers TH (lower left) and serotonin (lower right) indicated a differentiated cell state. In addition, the population of EGFP-only positive neurons was analyzed (upper right). Bar: 10 µm. B. Compared to wild type cells *Mecp2* ^−/y^ tEG nuclei (n = 100 each) revealed significantly more chromocenters, here visualized in whisker-box plots. Median values are shown as horizontal lines within the box, whiskers represent the 5–95 percentile, mean values are indicated as crosses, and outliers are visualized as dots. All analyzed samples show a highly significant (p<0.0001) difference of mean values. GFAP^+^/EGFP^−^ wild type cells revealed an average chromocenter number of 5.8±2.4 compared to 10.2±2.3 chromocenters in *Mecp2* ^−/y^ tEG cells. The mean chromocenter number per nucleus in TH^+^/EGFP^−^ *Mecp2* wild type cells was 5.9±2.0 versus 10.4±2.3 chromocenters in *Mecp2* deficient cells. In Sero^+^/EGFP^+^ wild type cells we found on average 4.9±1.4 chromocenter versus 9.3±2.0 chromocenters per nucleus in *Mecp2* deficient cells. The EGFP^+^ only population, showing 5.4±2.0 chromocenters in *Mecp2* wild type cells and 10.1±2.2 chromocenters in *Mecp2* ^−/y^ tEG cells, mirrored these tendencies. C. Unbiased analysis of data from three biological replicates (n = 100 per data set) for *Mecp2* wild type (solid line) and *Mecp2* deficient (dashed line) cells at different time points revealed significant decrease of chromocenters within the first two weeks of differentiation whereas extended differentiation time to 21 days did not result in further large scale heterochromatin remodeling. Error bars: 95% confidence intervals according to the mixed linear model.

### Statistical Evaluation

After chromocenter counting, data were analyzed using the software numbers (Apple, iWork, USA) and GraphPad Prism version 5.0. (GraphPad Software, USA, “www.graphpad.com”). Calculations and plots were done with Prism, if not stated otherwise. Whisker-box plots show the 5–95% confidence interval as whiskers, median values as horizontal lines, and indicate mean values as crosses and outliers as dots. P-values for significant differences of means (p<0.05) were calculated using an unpaired t-test (two-tailed) with Welch’s correction.

Concerning the unbiased counting approach the mean values of three independent biological replica sets were calculated and the effects of the variables ‘type’ (referring either to *Mecp2* ^wt^ or *Mecp2* ^−/y^) and ‘time’ (referring to differentiation time in days in LIF-free differentiation medium) on the dependent variables (i.e. chromocenter numbers) were analyzed using mixed linear models (SAS 9.2 PROC GLIMMIX; SAS Institute Inc., Cary,NC, USA) [65], [66]. We compared 13 covariance structures and selected a banded Toeplitz matrix (one band) according to the corrected Akaike criterion (AICC; [67]). For the calculation of degrees of freedom, we selected the Kenward-Roger approximation [65], [68]. The studentized residuals and conditional studentized residuals were examined for normality by means of graphical display (histograms and quantile residuum plots); nearly Gaussian distributions could be ascertained. For post hoc multiple comparisons we used Tukey adjusted tests.

## Results and Discussion

### Large Scale Heterochromatin Reorganization Takes Place during Embryonic Stem Cell Differentiation

To investigate the role of MeCP2 as a heterochromatin reorganizer we studied heterochromatin structure during neural differentiation (Figure 1) in *Mecp2* wild type ES cells (*Mecp2* ^wt^ tES) using the established mouse TK23 embryonic stem cell line [60], [62], [63]. In parallel, we generated a novel *Mecp2* deficient ES cell line (*Mecp2* ^−/y^ tEG) by knocking in *EGFP* under the neuronal tau promoter control (Figure S1A) to ascertain possible effects of *Mecp2* deficiency on heterochromatin reorganization. The *Mecp2* ^−/y^ tEG ES cell clones obtained were checked for correct targeting and genomic stability (Figure S1). *Mecp2* ^−/y^ tEG and *Mecp2* ^wt^ tEG stem cells were differentiated (Figure 1; Figure S2–S3) using a single step neural *in vitro* differentiation protocol [59]. This approach combined the advantage of a feeder free stem cell culture with neuronal specific EGFP reporter expression driven by the *tau* promoter and allowed us to focus directly on developing *Mecp2* wild type or *Mecp2* deficient neural cells.

We first evaluated heterochromatin remodeling during ES cell differentiation. In the presence of differentiation medium and upon leukemia inhibitory factor (LIF) withdrawal, *Mecp2* wild type ES cells start to differentiate as described by Ying *et al.* [62] and give rise to a variety of cell types including astroglia positive for glial fibrillary acidic protein (GFAP^+^) and neurons positive for EGFP (EGFP^+^) (Figure 1B). First morphological signs of neural differentiation became visible approximately one week after LIF withdrawal in form of rosette structures (Figure 1A). It is well documented that those rosettes are products of neural precursor cells and able to differentiate further into functional neurons [59], [62], [69], [70]. In our *in vitro* differentiation experiments, we found EGFP^+^ cells to be absent in undifferentiated cultures and early differentiation stages (day 0–3). In agreement with Ying *et al.* [62] we detected the onset of *EGFP* reporter expression after one week (day 5–7) indicating neural *tau* promoter activity and differentiation. In parallel EGFP^+^ cells exhibit a characteristic neural morphology and could already be identified as neurons by phase contrast microscopy. In the following days, we found the majority of EGFP^+^ cells located inside and nearby rosettes (Figure 1B, Figure S2). We also observed additionally scattered single EGFP^+^ cells throughout the culture (Figure S2B). Therefore, we assume that neural differentiation is not exclusively restricted to rosettes, although we cannot exclude cell migration out of those structures. After two weeks, approximately 60% of a differentiated culture showed tissue-like growth and former rosettes had grown into dense, multilayered islands (Figure 1A, Figure S1B). Almost all of those islands were found to consist of or at least contain high amounts of EGFP^+^ neurons (Figure 1B). Occasionally we observed nerve like connections between those islands that often were surrounded by GFAP^+^ astroglia (Figure 1B, Figure S2B). After three weeks, the amount of EGFP^+^ cells further increased and the former empty spaces were filled with cell monolayers or outgrowing axons. *Mecp2* ^−/y^ tEG cells behaved in a similar way (Figure S3) and we did not observe obvious differences between *Mecp2* wild type and *Mecp2* deficient cultures concerning cellular morphology and onset of differentiation. Multiple independent differentiation experiments yielded similar results, indicating that this system is a robust and simplified *in vitro* model for neural differentiation. Importantly, this system allowed us to follow differentiation of *Mecp2* wild type and *Mecp2* deficient ES cells into astroglia and neuronal cells, that are easy to discriminate by intrinsic or extrinsic markers and accessible for microscopic evaluation (Figure 1B).

Different studies have shown that the MeCP2 protein level increases with progressive differentiation in both neural [40], [71]–[74] and myogenic [5], [14] context. Moreover increasing MeCP2 protein level was linked to large-scale heterochromatin reorganization [5]. In different studies this reorganization was monitored by quantitative analysis of chromocenters [5], [6], [75] and has been shown to get disturbed by dysfunctional MeCP2 [76]. Given the known pathology of MeCP2, its high expression levels in differentiated neurons [40], [44], [46], [77] and its role in heterochromatin organization [54] we proceeded to quantitatively address effects of MeCP2 on heterochromatin architecture during neural *in vitro* differentiation. We performed at least three independent differentiation experiments with both *Mecp2* ^wt^ and *Mecp2* ^−/y^ tEG cultures. To cover different differentiation stages we acquired three-dimensional confocal images of nuclei at day 0 (undifferentiated cells), day 7 (early differentiation), day 13 (differentiated cell) and after day 20 (late differentiation). Since neither MeCP2 nor EGFP was detectable in IF experiments of early stages, we decided for experimental consistency to quantify heterochromatin organization data in biological replicas of 100 DAPI stained nuclei per time point (Figure 1C, Figure S4). Since we found MeCP2 to be always co-localized with DAPI intense peaks in EGFP^+^ cells as of day 13 (Figure 2), we consider both signals to be equivalent markers for quantification of heterochromatin organization [22].

By manual data evaluation and subsequent statistical analysis we found the chromocenter number in undifferentiated *Mecp2* wild type stem cells to scatter around 11±3.3 chromocenters per nucleus (Figure 1C, Figure S4, S5). One week after LIF withdrawal the chromocenter average decreased significantly to 7.3±2.2 while the overall scatter got slightly reduced (Figure S5). This trend continues and after two weeks the average chromocenters number per nucleus was 5.3±1.7. These data also agree with recent results reported by Singleton et al. [55] for *Mecp2* wild type primary cortical mouse neurons. We also prolonged our differentiation studies up to day 23 but did not detect further decrease and found both average and standard deviation to remain constant (5.3±1.7). Testing the mean values of the early and late differentiated data sets for significant differences yielded a p-value of 0.177, confirming that both sets were not significantly different.

These findings agree with previous reports on neural [6], [78] and myogenic [5] *in vitro* differentiation. Although the MeCP2 protein level in muscle is not as high as in brain tissue where the levels are the highest [40], [41], [71], we previously observed MeCP2 increase during myoblast differentiation, which was sufficient to reorganize heterochromatin [5]. This suggests that heterochromatin reorganization is a shared feature of multiple differentiation pathways and correlated with MeCP2 protein level whose threshold is though cell lineage specific. It would be interesting to compare MeCP2 thresholds during differentiation of controlled and simplified *in vitro* systems such as the one presented here and their more complex *in vivo* counterparts, which not only respond to spatial and temporal cues but also to environmental stimuli.

### MeCP2 becomes Detectable in Late Differentiation Stages and is Associated with Chromocenters

Previous studies linked heterochromatin organization to MeCP2 protein level in different cell lines [5], [6]. Accordingly, we found a significant increase of MeCP2 signal in differentiated cells (Figure 2B) as we compared undifferentiated (day 0) to differentiated *Mecp2* wild type ES cells (day 13) in immunoblots and immunofluorescence experiments. In both assays, *Mecp2* ^−/y^ tEG controls revealed no detectable MeCP2 signals (Figure 2). With a rat monoclonal antibody [64] we were able to show a maximum of MeCP2 signals in differentiated EGFP^+^ neurons as of day 13 (Figure 2A). Additional line scan profiles in optical sections further showed a co-localization of MeCP2 and DAPI signal as reported by Nan et al. [22] at chromocenters sites in all EGFP^+^ cells observed. In contrast, MeCP2 line scans of EGFP^+^ cells in *Mecp2* ^−/y^ tEG cultures remained within background noise.

Interestingly, our rat monoclonal antibody against MeCP2 also detected a relatively weak but positive MeCP2 signal in GFAP^+^ astroglia as of day 13 compared to the signal intensity in neurons (Figure 2A). As MeCP2 was initially reported to be absent in glial cells [40], [41], [71] our data contribute to the increasing evidence arguing for MeCP2’s engagement in glia [44], [46], [47], [79], [80]. Moreover, we did not find all GFAP^+^ cells to be positive for MeCP2. This observation suggests a temporally restricted presence and function of MeCP2 in the astroglia lineage, which could also explain the contradicting data on presence or absence of MeCP2 in this lineage.

### The Absence of MeCP2 during Neural *in vitro* Differentiation does not Interfere with Differentiation into a Variety of Neural Subtypes and Astroglia

It has been shown that MeCP2 is able to cluster [54] and to reorganize heterochromatin in a dose-dependent manner and that an increase in MeCP2 protein results in a decrease of average chromocenter numbers [5]. It is also known that during neural differentiation MeCP2 protein level increases to reach a maximum in differentiated neurons [32]. Therefore, we wanted to know how the lack of MeCP2 might interfere with differentiation of ES cells. Would *Mecp2* deficiency preserve an undifferentiated chromatin organization state and perhaps interfere with the capability to differentiate? To address this question we differentiated *Mecp2* ^−/y^ tEG stem cells by LIF deprivation for 13 days and checked for the presence of astroglia and neural differentiation markers as a control for general differentiation capability (Figure 3). We found both *Mecp2* wild type and *Mecp2* deficient stem cells positive for the astroglia marker GFAP (glial acidic fibrillary protein) and the neural markers tyrosine hydroxylase (TH) and serotonin (Sero). Therefore, we conclude that the lack of MeCP2 in *Mecp2* ^−/y^ tEG does not interfere with differentiation capacity *per se*. Moreover, we did not observe obvious differences concerning the amount of marker positive cells nor the timing of marker expression.

While GFAP and EGFP signals were mutually exclusive and positive cells constitute separate populations, we could subdivide EGFP^+^ neurons into EGFP^+^ only and EGFP^+^/marker^+^ populations. However, it remains to be answered if the EGFP^+^ only population could be further subdivided or resembles an earlier differentiation state before the onset of both tyrosine hydroxylase or serotonin marker expression.

After showing that both cell lines were able to produce differentiated neurons and astroglia, our next step was a quantitative analysis of heterochromatin reorganization in differentiated cells. If heterochromatin reorganization would be truly MeCP2 dependent, *Mecp2* deficiency would prevent a decrease of average chromocenters number per nucleus during differentiation.

### MeCP2 Deficiency Causes Structural Changes in Heterochromatin Organization of Neurons and Astroglia

As we analyzed differentiated *Mecp2* wild type stem cells at day 13 we found the average chromocenter number in EGFP^+^ cells to be significantly decreased compared to undifferentiated cells (Figure 4). In EGFP^+^/TH^+^ wild type neurons we observed an average number of 5.9±2 chromocenter per nucleus compared to 10.4±2.3 chromocenter in *Mecp2* deficient cells (Figure 4B). These numbers were quite similar in the corresponding EGFP^+^ only population showing 5.4±2 chromocenters in wild type and 10.1±2.2 chromocenters in *Mecp2* deficient cells. Also EGFP^+^/Sero^+^ neurons revealed a significant decrease of chromocenters in differentiated cells: while *Mecp2* wild type cells showed 4.9±1.4 chromocenters at day 13, *Mecp2* deficient cells exhibit at the corresponding time point 9.3±2.0 chromocenters. In addition to neuronal cells we also analyzed the GFAP^+^ astroglia population. Here we found 5.8±2.4 chromocenters in *Mecp2* wild type cells compared to 10.2±2.3 chromocenters in *Mecp2* deficient cells.

It is noteworthy that although MeCP2 protein levels do differ in neurons (EGFP^+^) and astroglia (GFAP^+^), we found similar tendencies concerning chromocenter numbers for both lineages, i.e., an increase in the absence of MeCP2 (Figure 4). As we have previously reported a concentration dependent effect of MeCP2 on heterochromatin reorganization [5], it is tempting to speculate that the threshold level of MeCP2 needed for chromatin remodelling varies for different lineages. Furthermore, recent evidence hint to distinct requirements for MeCP2 at different developmental time windows [59]. Comparative systematic analyses (time and concentration) of additional parameters such as DNA methylation, other MBD proteins and additional known chromatin remodelers in relevant brain regions and in other *in vitro* differentiation systems will be required to clarify this issue. In addition, it would be very interesting to investigate chromatin reorganization in differentiating ES cells derived from heterozygous *Mecp2^−/x^* mice. The latter would allow direct comparison within the same culture of MeCP2 positive and negative cells, thus reducing any potential differences related to cellular heterogeneity.

We conclude that the differences in chromocenter clustering observed between *Mecp2* wild type and *Mecp2* ^−/y^ tEG cells were not due to a lack of differentiation capability of *Mecp2* deficient cells. Hence, we assume that MeCP2 is necessary for heterochromatin reorganization and responsible for chromocenter clustering in astroglia and neuronal lineages. We could confirm our hypothesis in an unbiased approach as we compared *Mecp2* wild type and deficient cells at different differentiation time points in biological replicas of 100 nuclei each. A mixed linear model analysis confirmed a highly significant influence of the parameters “type” (i.e. MeCP2 protein presence) and “time” (i.e. differentiation in days) on the mean chromocenter number as well as an interaction effect of both parameters within a data set (Figure 4C). Although undifferentiated *Mecp2* wild type and deficient cells were quite similar (wt: 11.8±0.8 versus −/y: 11.2±0.3 chromocenter per nucleus; Figure S5), significant differences in heterochromatin reorganization became apparent after 7, 13 and 20 days. Within the first two weeks of differentiation the average number dropped in *Mecp2* wild type cells to 5.3±0.5 chromocenters while the numbers in *Mecp2* deficient cells stayed elevated at 9.9±0.6 chromocenters. The post hoc test showed that the difference between day 0 and day 13 was highly significant for *Mecp2* wild type and significant for *Mecp2* deficient cells. Since it was possible that *Mecp2* deficient cells were just delayed in heterochromatin remodeling we extended our analysis to day 21. At that time point we observed 5.1±0.3 chromocenters in *Mecp2* wild type cells and 9.7±0.2 chromocenter in *Mecp2* ^−/y^ tEG cells. Both cell lines did not show a significant difference compared to the previous differentiation time point. Hence, we assume that after two weeks of *in vitro* differentiation chromocenter numbers remain quite constant and the observed differences in *Mecp2* ^−/y^ tEG cells are not due to a general differentiation delay. During *in vivo* brain development however, signal gradients, positional information, neighborhood, and neuronal activity, etc. might contribute further clues too complex to be monitored by an *in vitro* culture model.

Our results support the notion of MeCP2 as a multifunctional and chromatin structure organizing factor. Given that the lack of MeCP2 seems not to be crucial for early differentiation [40], and results in only mild transcriptional changes [24], [26], we propose that MeCP2 is rather a key protein for stabilization and maintenance of the differentiated heterochromatin structure. Further work will focus on elucidating the role of MeCP2 regulated heterochromatin compartments as transcriptional silencing and/or trapping compartments.

## Supporting Information

Figure S1 **Targeting of** ***EGFP*** **in the** ***tau*** **locus produces ES cells stably expressing** ***EGFP*** **in postmitotic neurons. A.** The schematic drawing modified according to Tucker *et al*. depicts the targeting strategy used to insert an *EGFP* cDNA into exon 1 of the *Mapt*/*tau* locus, resulting in the expression of EGFP protein under control of the neuronal *Maptt*/*tau* promoter. The integrated cassette consist of an *EGFP* cDNA sequence (green), located upstream of a *Pgk-Neo^r^* resistance cassette (*Neo^R^*). Indicated are *BamH*I restriction sites, exon 1 integration site (black), and the 5′ and 3′ genomic arms for homologous recombination in light and dark grey, respectively. Blue and red arrows mark the position of the primers used for the amplification reactions shown in B (left and right panel, respectively). **B.** Long-range amplification (left) shows some representative transfected clones (lanes 3–10) The 2.5 kb band indicates the correct insertion of *EGFP* in *tau* locus in clones B9, C3, C9, D1 and D11. A TK23 sample and a wild-type sample have been also included as a positive and negative control, respectively (lanes 1,2). Inverse PCR results (right) show amplification of some representative transfected clones (lanes 5–10). The strategy allows the amplification of the genomic region flanking the *EGFP*/*Neo* ^R^ cassette. Only clones carrying the cassette in *tau* locus will produce a band of expected size (1085 bp). A 46C sample (derived from a cell line where the same targeting vector has been used to knock in *EGFP* cDNA in *Sox2* promoter) and a TK23 sample have been also included as a positive and negative control, respectively (lanes 3,4). **C.** Representative image of a Giemsa-stained metaphase spread from one of the clones whose targeting has been positively verified. Karyotype analysis shows no obvious chromosomal aberrations.
(TIF)

Figure S2 **The majority of neurons develop from rosettes and from interconnected clusters over time. A.** Shown is a typical multilayered rosette region in a differentiated *Mecp 2* wild type ES culture (upper right, see also Figure 1). For the indicated region of interest (dashed box) inverted single channel images (left) are shown as well as merged channel images (lower right). Neurons are identified by *tau* promotor driven *EGFP* (green) and astroglia are visualized by anti glial fibrillary acidic protein (GFAP) antibody (red). EGFP and GFAP signals are mutually exclusive and mark different cell populations. While the majority of EGFP^+^ cells reside inside rosettes some single EGFP^+^ neurons are located outside rosettes. GFAP^+^ cells are excluded from the center of rosettes but often found adjacent to outer rosette regions. Bar: 10 µm. **B.** After two weeks of LIF deprivation tissue-like regions (upper left corners) and multilayered islands (asterisks) appear throughout *Mecp2* wild type and *Mecp2* deficient cultures. Most of those islands consisted of EGFP^+^ cells. In addition single EGFP^+^ cells (arrow head) are found in less dense regions. Occasionally EGFP^+^ islands are connected by nerve like fibers, as shown in the live cell image. Bar: 50 µm.
(TIF)

Figure S3 ***Mecp2*** **^−/y^ tEG cells revealed no obvious morphological differences compared to** ***Mecp2*** **^wt^ tEG** ***in vitro*** **differentiation. A.** Shown are DAPI DNA stainings (blue) and phase contrast (Ph) images of undifferentiated (day 0), early differentiated (day 7), differentiated (day13), and late differentiated (>20 days) *Mecp2* ^−/y^ tEG cells. Similar to *Mecp2* ^wt^ cultures, first morphological signs of neural differentiation are rosette structures, clearly visible as of day 7. *Tau* promotor driven *EGFP* reporter expression shortly thereafter could verify neural fate. Bar: 10 µm. **B.** As in *Mecp2* ^wt^ cultures the majority of EGFP positive cells is found inside (arrow head) or in the vicinity of rosettes. Bar: 10 µm.
(TIF)

Figure S4 **Biological replicates demonstrate the robustness of the differentiation system.** The whisker-box-plots show a comparison of three biological replica experiments based on DAPI signals (set A-C; n = 100 each) and revealed a quite robust data distribution. Results for *Mecp2* wild type (*Mecp2* ^wt^) cells are shown on the left; for *Mecp2* deficient (*Mecp2* ^−/y^tEG) cells on the right. Horizontal lines depict median values, crosses indicate mean values, outliers are depicted as dots, and whiskers indicate the 5–95 percentile.
(TIF)

Figure S5 **Differences in heterochromatin organization of** ***Mecp2*** **wild type and deficient cells during differentiation. A.** Comparison of *Mecp2* wild type (*Mecp2* ^wt^ tEG; left) and *Mecp2* deficient (*Mecp2* ^−/y^ tEG; right) cells revealed significant differences in heterochromatin reorganization over differentiation. While in *Mecp2* wild type cells the mean chromocenter number per nucleus (based on DAPI signal) halves from 11.8 (±0.8) to 5.1 (±0.3), it remains elevated in *Mecp2* deficient cells and only slightly drops from 11.2 (±0.3) to 9.9 (±0.6). A Scatter plots mark the mean value as red lines within a 95% confidence interval (black whiskers) for each time point. **B.** Accompanying whisker-box-plots indicate highly significant differences (p<0.0001) between data sets (asterisks). For both *Mecp2* wild type (right) and *Mecp2* deficient cells (left) no significant differences of mean and median values were detected between late differentiation stages (day 13 and >20 days).
(TIF)
