# Supplementary figures and images for: MeCP2 Dependent Heterochromatin Reorganization during Neural Differentiation of a Novel Mecp2-Deficient Embryonic Stem Cell Reporter Line
Source: PLoS One. 2012 Oct 24;7(10):e47848. doi: 10.1371/journal.pone.0047848 (PMC3480415; doi:10.1371/journal.pone.0047848)

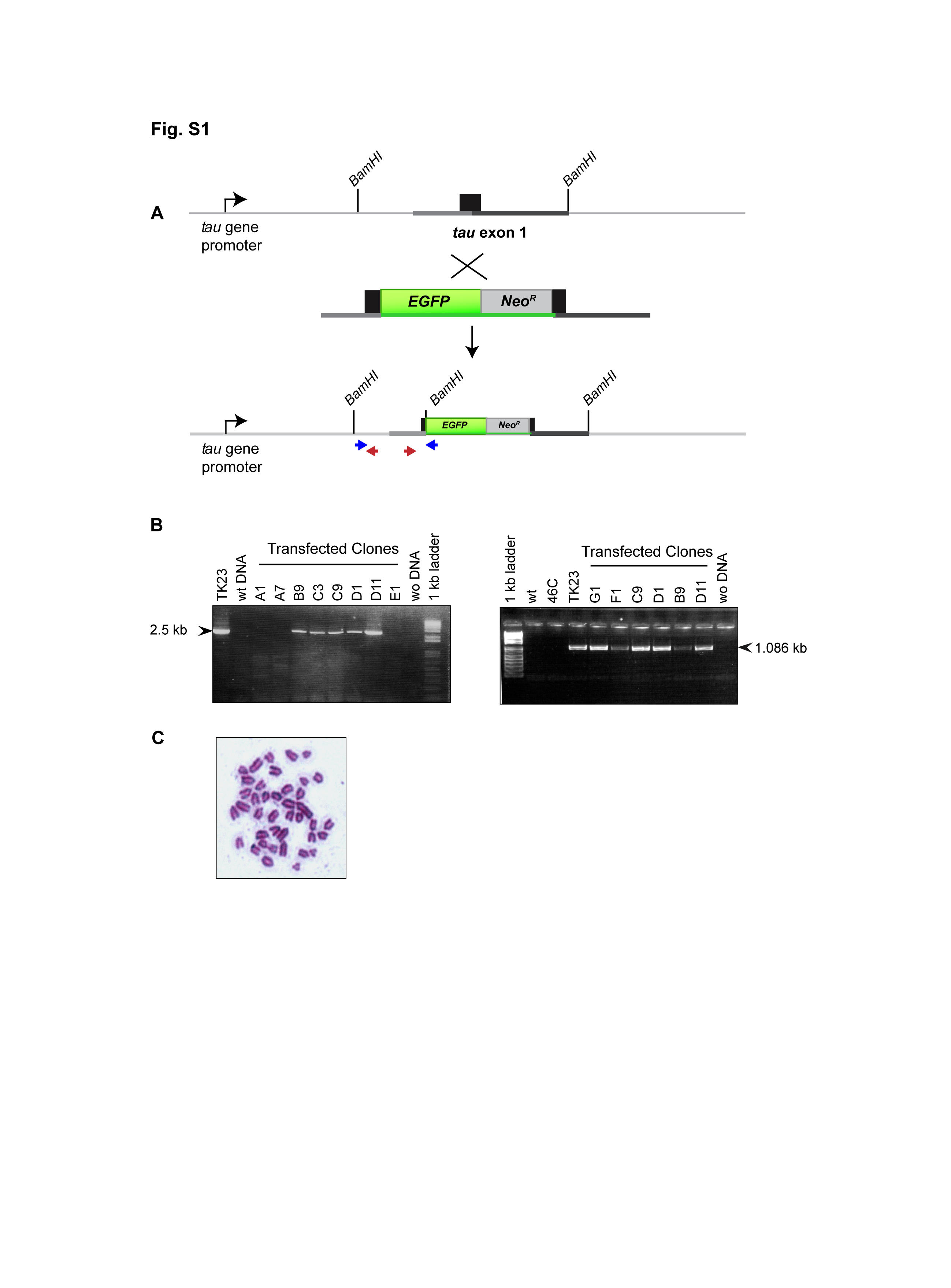

Supplement: Figure S1 — Targeting of EGFP in the tau locus produces ES cells stably expressing EGFP in postmitotic neurons. A. The schematic drawing modified according to Tucker et al. depicts the targeting strategy used to insert an EGFP cDNA into exon 1 of the Mapt/tau locus, resulting in the expression of EGFP protein under control of the neuronal Maptt/tau promoter. The integrated cassette consist of an EGFP cDNA sequence (green), located upstream of a Pgk-Neor resistance cassette (NeoR). Indicated are BamHI restriction sites, exon 1 integration site (black), and the 5′ and 3′ genomic arms for homologous recombination in light and dark grey, respectively. Blue and red arrows mark the position of the primers used for the amplification reactions shown in B (left and right panel, respectively). B. Long-range amplification (left) shows some representative transfected clones (lanes 3–10) The 2.5 kb band indicates the correct insertion of EGFP in tau locus in clones B9, C3, C9, D1 and D11. A TK23 sample and a wild-type sample have been also included as a positive and negative control, respectively (lanes 1,2). Inverse PCR results (right) show amplification of some representative transfected clones (lanes 5–10). The strategy allows the amplification of the genomic region flanking the EGFP/Neo R cassette. Only clones carrying the cassette in tau locus will produce a band of expected size (1085 bp). A 46C sample (derived from a cell line where the same targeting vector has been used to knock in EGFP cDNA in Sox2 promoter) and a TK23 sample have been also included as a positive and negative control, respectively (lanes 3,4). C. Representative image of a Giemsa-stained metaphase spread from one of the clones whose targeting has been positively verified. Karyotype analysis shows no obvious chromosomal aberrations. (TIF) [file pone.0047848.s001.tif]

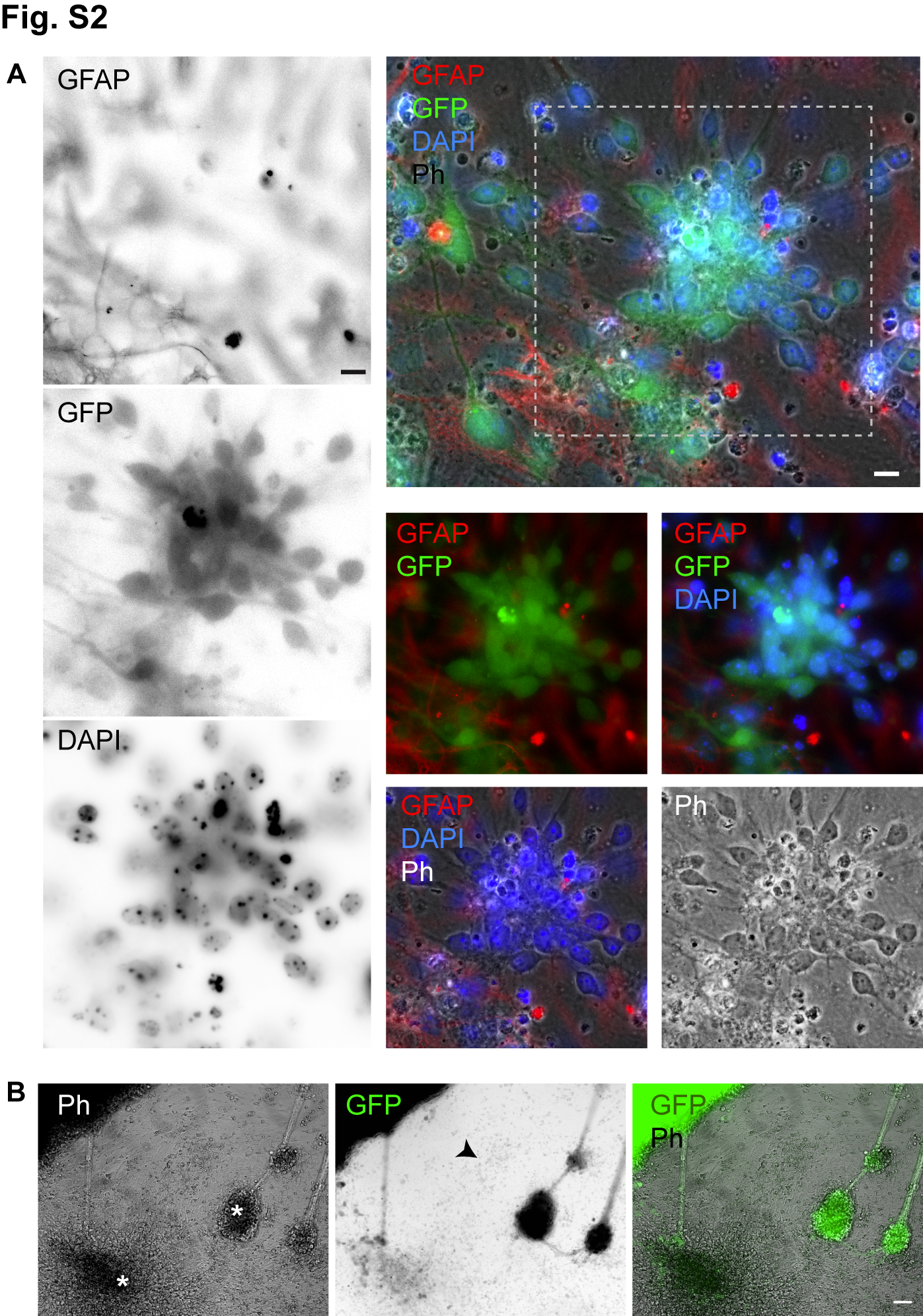

Supplement: Figure S2 — The majority of neurons develop from rosettes and from interconnected clusters over time. A. Shown is a typical multilayered rosette region in a differentiated Mecp 2 wild type ES culture (upper right, see also Figure 1). For the indicated region of interest (dashed box) inverted single channel images (left) are shown as well as merged channel images (lower right). Neurons are identified by tau promotor driven EGFP (green) and astroglia are visualized by anti glial fibrillary acidic protein (GFAP) antibody (red). EGFP and GFAP signals are mutually exclusive and mark different cell populations. While the majority of EGFP+ cells reside inside rosettes some single EGFP+ neurons are located outside rosettes. GFAP+ cells are excluded from the center of rosettes but often found adjacent to outer rosette regions. Bar: 10 µm. B. After two weeks of LIF deprivation tissue-like regions (upper left corners) and multilayered islands (asterisks) appear throughout Mecp2 wild type and Mecp2 deficient cultures. Most of those islands consisted of EGFP+ cells. In addition single EGFP+ cells (arrow head) are found in less dense regions. Occasionally EGFP+ islands are connected by nerve like fibers, as shown in the live cell image. Bar: 50 µm. (TIF) [file pone.0047848.s002.tif]

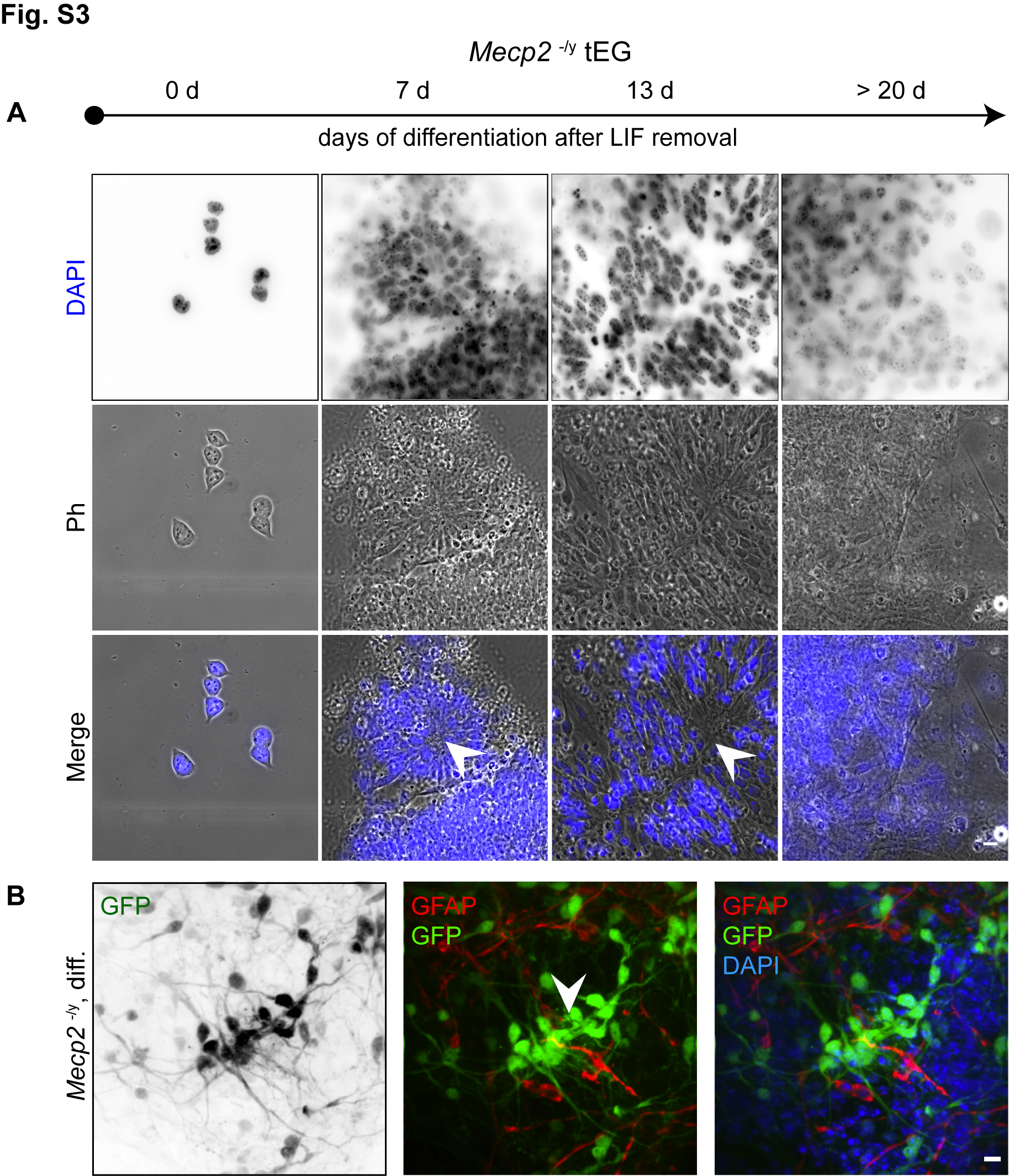

Supplement: Figure S3 — Mecp2 −/y tEG cells revealed no obvious morphological differences compared to Mecp2 wt tEG in vitro differentiation. A. Shown are DAPI DNA stainings (blue) and phase contrast (Ph) images of undifferentiated (day 0), early differentiated (day 7), differentiated (day13), and late differentiated (>20 days) Mecp2 −/y tEG cells. Similar to Mecp2 wt cultures, first morphological signs of neural differentiation are rosette structures, clearly visible as of day 7. Tau promotor driven EGFP reporter expression shortly thereafter could verify neural fate. Bar: 10 µm. B. As in Mecp2 wt cultures the majority of EGFP positive cells is found inside (arrow head) or in the vicinity of rosettes. Bar: 10 µm. (TIF) [file pone.0047848.s003.tif]

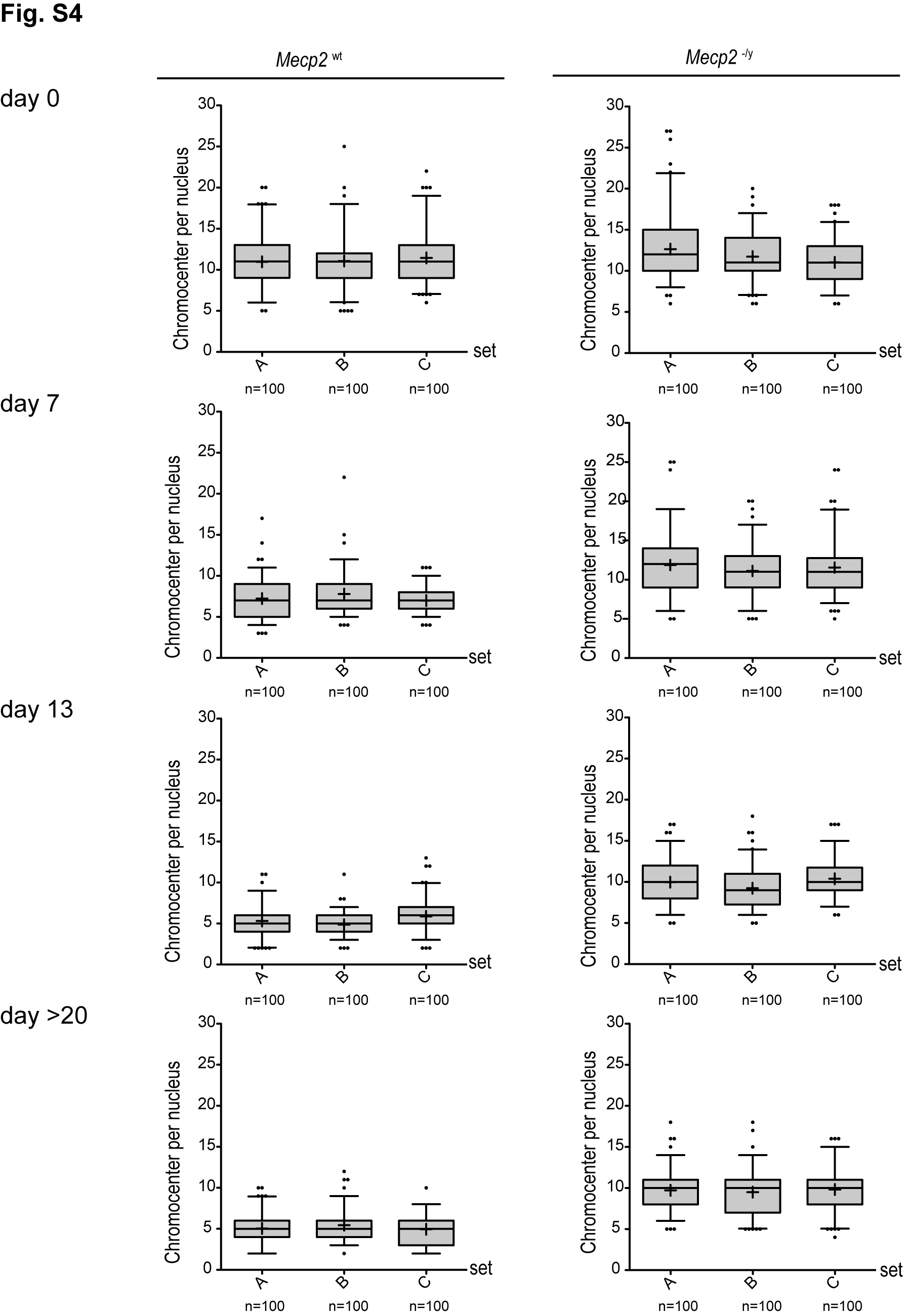

Supplement: Figure S4 — Biological replicates demonstrate the robustness of the differentiation system. The whisker-box-plots show a comparison of three biological replica experiments based on DAPI signals (set A-C; n = 100 each) and revealed a quite robust data distribution. Results for Mecp2 wild type (Mecp2 wt) cells are shown on the left; for Mecp2 deficient (Mecp2 −/ytEG) cells on the right. Horizontal lines depict median values, crosses indicate mean values, outliers are depicted as dots, and whiskers indicate the 5–95 percentile. (TIF) [file pone.0047848.s004.tif]

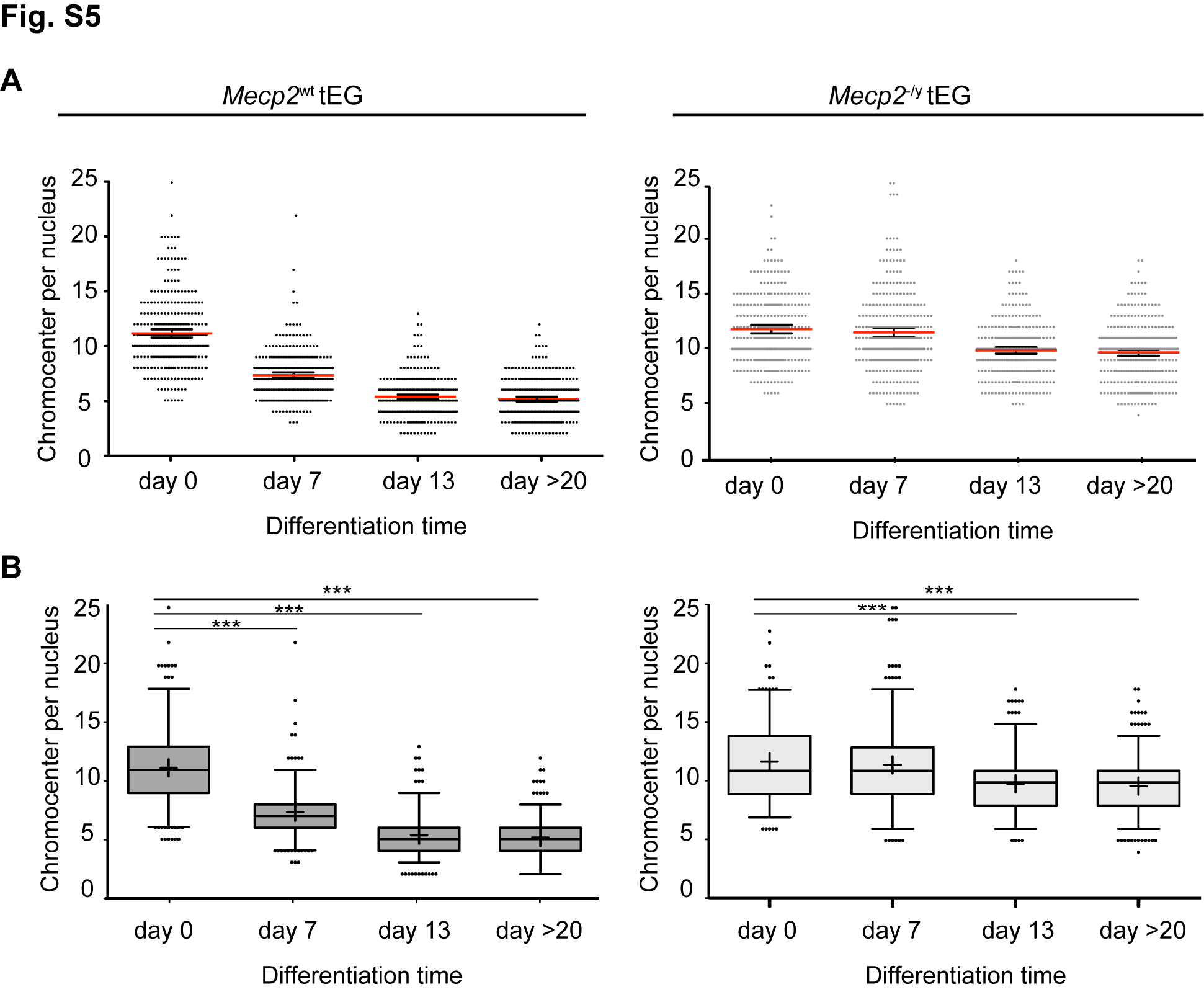

Supplement: Figure S5 — Differences in heterochromatin organization of Mecp2 wild type and deficient cells during differentiation. A. Comparison of Mecp2 wild type (Mecp2 wt tEG; left) and Mecp2 deficient (Mecp2 −/y tEG; right) cells revealed significant differences in heterochromatin reorganization over differentiation. While in Mecp2 wild type cells the mean chromocenter number per nucleus (based on DAPI signal) halves from 11.8 (±0.8) to 5.1 (±0.3), it remains elevated in Mecp2 deficient cells and only slightly drops from 11.2 (±0.3) to 9.9 (±0.6). A Scatter plots mark the mean value as red lines within a 95% confidence interval (black whiskers) for each time point. B. Accompanying whisker-box-plots indicate highly significant differences (p<0.0001) between data sets (asterisks). For both Mecp2 wild type (right) and Mecp2 deficient cells (left) no significant differences of mean and median values were detected between late differentiation stages (day 13 and >20 days). (TIF) [file pone.0047848.s005.tif]
